# Interaction with gravitropism, reversibility and lateral movements of phototropically stimulated potato shoots

**DOI:** 10.1007/s10265-016-0821-4

**Published:** 2016-03-31

**Authors:** D. Vinterhalter, J. Savić, M. Stanišić, Ž. Jovanović, B. Vinterhalter

**Affiliations:** 1 Institute for Biological Research, University of Belgrade, Bulevar Despota Stefana 142, 11060 Belgrade, Serbia; 2 Institute of Molecular Genetics and Genetic Engineering, University of Belgrade, P.O. Box 23, 11 010 Belgrade, Serbia

**Keywords:** Gravitropism, Phototropism, Reversibility, *Solanum tuberosum* L., Tropic interactions

## Abstract

Phototropic (PT) and gravitropic (GT) bending are the two major tropic movements that determine the spatial position of potato shoots. We studied PT bending of potato plantlets grown under long-day photoperiods in several prearranged position setups providing different interactions with the GT response. Starting with the standard PT stimulation setup composed of unilateral irradiation of vertically positioned shoots, experiments were also done in antagonistic and synergistic setups and in treatments with horizontal displacement of the light source. In the standard setup, PT bending suppressed the GT bending, which could occur only if the PT stimulation was cancelled. The antagonistic position, with phototropism and gravitropism attempting to bend shoots in opposite directions, showed phototropism and gravitropism as independent bending events with the outcome varying throughout the day reflecting diurnal changes in the competence of individual tropic components. Whilst gravitropism was constant, phototropism had a marked daily fluctuation of its magnitude with a prominent morning maximum starting an hour after the dawn in the growth room and lasting for the next 6 h. When phototropism and gravitropism were aligned in a synergistic position, stimulating shoot bending in the same direction, there was little quantitative addition of their individual effects. The long period of morning PT bending maximum enabled multiple PT bending events to be conducted in succession, each one preceded by a separate lag phase. Studies of secondary PT events showed that potato plantlets can follow and adjust their shoot position in response to both vertical and horizontal movements of a light source. PT bending was reversible, since the 180° horizontal change of a blue light (BL) source position resulted in reversal of bending direction after a 20-min-long lag phase.

**Electronic supplementary material:**

The online version of this article (doi:10.1007/s10265-016-0821-4) contains supplementary material, which is available to authorized users.

## Introduction

Potato plantlets propagated in vitro by single node explant subculturing have been recently employed to study tropic responses of dicotyledonous plants (Vinterhalter et al. 2012, 2015). Grown under long-day (16 h light/8 h dark) conditions, these plantlets performed vigorous PT and GT bending movements. PT and GT bending magnitudes were subjected to regular daily changes in accordance with a specific diurnal rhythm. An hour after the lights in the growth chamber were turned on (dawn at 7.00 h), the PT response reached its daily maximum, which lasted through the next 6–7 h, declining in the afternoon and then staying low during the night. Diurnal changes of the GT response magnitude were less prominent, but they alternated with the PT response changes with highest values at dawn, late in the afternoon and during the night.

Detection of diurnal changes in the magnitude of PT and GT responses forced us to abandon the classic view of the PT response as a constant and predictable feature of etiolated or de-etiolated seedlings. In light-grown plantlets, the PT response showed a dynamic, time-related dimension; therefore, it was considered as competence (capacity)—subject to regular daily changes (Vinterhalter et al. 2015).

PT and GT bending capacities of plants are tightly connected. Plant organs often grow at specific angles in relation to the vertical, called gravitational set-point angles (Digby and Firn 1995). These are equilibrium points at which organs show no gravity-induced differential growth (Hangarter 1997). Since the gravity on Earth is omnipresent and unavoidable, the GT stimulation, with its corresponding bending response, needs to be studied and accounted for as a force constantly modifying the PT competence. Cancelling the GT response by the use of clinostats, random positioning machines or microgravity has its own merits and advantages in experimental studies. However, PT and GT responses with their opposite action in shoots seem to be just two sides of the same coin, and they need to be studied in parallel. Interactions of PT and GT bending forces can be best studied in positional setup trials enabling their antagonistic and synergistic actions, as described by Franssen (1980) for sunflower. *Arabidopsis thaliana* studies done on flower stalks by Fukaki et al. (1996a, b) showed that gravitropism is superior to phototropism, which is the same as in sunflower (Franssen 1980), cress (Hart and Macdonald 1981) and pea (Britz and Galston 1982). We therefore investigated PT versus GT interactions in potato, comparing them to findings known for other plant species.

Studies that we have previously performed on potato offered many new insights in the tropic response relations. The use of in vitro culture techniques enables complete and accurate control over the experimental conditions in combination with minimal interaction with the studied material. Once the nodal explants are enclosed in their culture vessels, all subsequent manipulations are done with no direct physical contact with plantlets. Culture flasks with their plantlets can be easily rotated, inclined or even overturned to horizontal positions as required for GT bending studies.

To an observer, PT and GT shoot bending may appear to arise from the action of some putative forces triggered by tropic stimulation. These forces appear through the lag phase after the start of tropic stimulation; they conduct or perhaps just accompany tropic bending processes and persist shortly after the tropic stimulation ends. Theses transient forces are virtual by nature, as they are not accompanied by appropriate expenditures of metabolic energy.

On the other hand, PT and GT stimulation are done by true physical forces. In contrast to gravitation, which is a strong, omnipresent force of constant intensity, the BL that we provided for PT stimulation can be highly variable in its intensity. In light-grown potato plantlets, the magnitude of the PT response was found to be directly proportional to the irradiation intensity (Vinterhalter et al. 2012), meaning that light is at least partly used to fuel PT bending. Furthermore, PT bending requires constant irradiation with BL. Light signals cannot be stored in standard conditions, and when the light is turned off, PT bending stops 20 min later (Vinterhalter and Vinterhalter 2012).

Latent (lag) phases of tropic bending are important features (Rich and Smith 1986); they can indicate the nature of the processes involved in the bending, as they contain the whole chain of signal transduction events. However, tracing the line of signal transduction, which is currently the main approach in tropic studies, will not bring us directly to the tropic response effector. According to Friml (2003), differential auxin distribution resulting in auxin gradients underlies a multitude of developmental processes in plants and tropic responses, and it is difficult to envision a common mechanism enabling a chemical compound as simple as auxin to act as a specific signal in such a variety of processes (Vanneste and Friml 2009). Thus, backtracking of the events and conditions leading to the beginning of the bending response could perhaps be used as an alternative approach in connecting effector activity with tropic signal transduction.

Auxin gradients are considered central events for the establishment of tropic curvatures (Esmon et al. 2006; Stone et al. 2008), especially in root gravitropism, where gradients are directly connected to the presence of PIN auxin efflux. In *Arabidopsis* root columellae, PIN3 protein was shown to reallocate after just 2 min of gravistimulation (Friml 2003). Increased auxin levels were also detected in GT- and PT-stimulated shoots and coleoptiles of various plant species (Tanaka et al. 2006).

There is no doubt that in all plants, phototropins 1 and 2 are the main receptors of light presence and of direction required for PT bending (Christie and Murphy 2013). In some species, including *Arabidopsis*, phytochromes and cryptochromes are also involved, as they modulate the magnitude of the PT response (Goyal et al. 2013; Whippo and Hangarter 2003), creating a network of interconnected pigment factors. However, in potato shoots, PT and GT bending responses are insensitive to the presence of red-, amber- and yellow-coloured light, indicating that PT bending regulation of potato is less complex than that in *Arabidopsis*.

As a model system, potato shoot cultures have certain advantages in comparison to studies done on etiolated seedlings. Among others, it is noteworthy that the long morning period of the high PT-bending-capacity characteristic for potato shoot cultures supports multiple PT events appearing in fast succession. Every new event of this succession starts also with a new lag phase. Therefore, it is possible to study and compare expression of lag phases arising in response to restarts of PT stimulation or to changes in the direction of the incoming PT-stimulating BL.

Studies of lag phases following lateral displacement of the light source indicated that potato plantlets are equipped to track both vertical and horizontal movements of the light sources, inducing PT stimulation. Therefore, data presenting kinetics of horizontal adjustment of shoot positions were also collected and added to this study. These studies also showed reversibility as an important feature of the potato PT response, enabling very fast changes of the shoot bending direction.

Data presented in this study show a high level of integration between the PT and GT bending responses of potato shoots. Although independent in signal perception and action development, PT and GT bending probably share the resources of the same effector mechanism which could be the one based on radial water translocation. The lateral PT shoot movement and PT bending reversibility further extend the remarkable PT bending resources available to potato plantlets.

## Materials and methods

Potato cultivar Désirée shoot cultures were grown and maintained on plant growth regulator-free Murashige and Skoog (MS) medium (Murashige and Skoog 1962) supplemented with 3 % sucrose and 0.7 % agar in 270 mL glass jars (*Φ* 62 × 107 mm) with 45–50 mL of medium and translucent polypropylene caps. Cultures were maintained in growth chambers by subculturing apical and single node explants at 3–4 week intervals according to the propagation procedure suggested by Hussey and Stacey (1981). Growth chambers were equipped with Philips TLD fluorescent lamps providing fluence rate of 70 µmol m^−2^ s^−1^ as measured by a LiCor 1400 spectrophotometer with a Quantum sensor. Temperature was maintained at 24 ± 0.5 °C under a long-day (16/8 h) light to darkness photoperiod. Same temperature was maintained in dark chambers during the tropic stimulation treatments.

Cultures intended for tropic treatment measurements were set only with single node explants. Apical bud explants were used to maintain and multiply cultures. Single node explants required 9–14 days in the growth chamber to produce plantlets with shoots reaching 40–55 mm, a height suitable for tropic treatments. At this stage, explants had well-developed adventitious roots and they were referred to as plantlets. Studies were conducted at the time of maximum morning phototropism, usually some 1–2 h after the beginning of the day in the growth chamber.

**Fig. 1:**
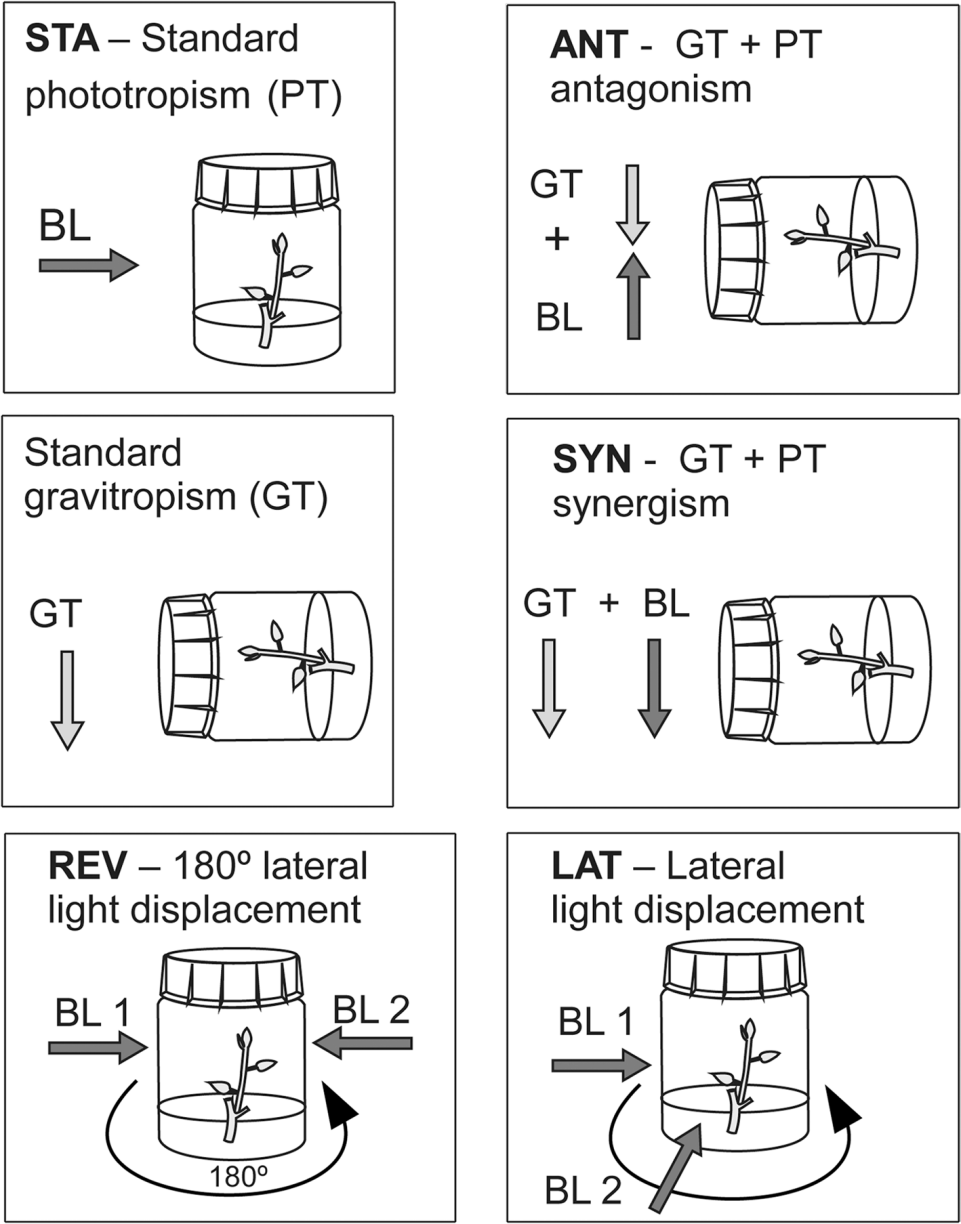
Positional setup arrangements used in the study. The standard position setup (STA) is the standard position of the phototropic (PT) stimulation used in most tropic studies. Culture flasks and plantlets within were in the* vertical position*, and the unilateral blue light (BL) of ~470 nm was coming at 24 μM m^−2^ s^−1^. Prior to PT stimulation, cultures were grown in a long-day growth room with fluorescent lamps positioned above the cultures, providing 70 μM m^−2^ s^−1^, 4500° K. Standard gravitropism was obtained by placing culture flasks with plantlets in a* horizontal position*. The antagonist position setup was obtained by placing the culture flasks with plantlets horizontally, and a BL source was positioned beneath the culture flasks illuminating cultures at 24 μM m^−2^ s^−1^. The synergistic setup position was also obtained by placing culture flasks with plantlets horizontally; only the BL source illuminates cultures from above. The lateral displacement setup (LAT) is a modification of the STA position in which some 50–60 min after the beginning of BL stimulation, the BL source was laterally displaced to some desired angle, such as 10°, 15°, 30°, 45°, 60° or 90° in relation to the original BL source position. The reversion position setup (REV) is a special case of LAT where the lateral displacement angle is 180°. *Arrows* indicate direction of tropic forces; shoots bend in the opposite direction. Lateral displacement was done by rotating the culture flasks in front of the BL source on a simplified turntable. REV and LAT positions have two distinct lag phases; the second one starts after the lateral light displacement

Three different PT stimulation setups (antagonistic, synergistic and standard) were used in the PT versus GT interaction studies, and a separate setup was used to study shoot position compensation following lateral displacement of the BL sources (Fig. 1). Light for the PT stimulation in all experimental setups was provided by Philips 1 W accent colour blue LED lamps (one lamp per culture flask), producing 24 µmol m^−2^ s^−1^ irradiance at the surface of the culture flasks. For the GT stimulation, flasks with cultures were turned to a horizontal position. Antagonistic (ANT) and synergistic (SYN) studies were done in a flask holder designed to support horizontally positioned culture flasks, which could then be irradiated from below (ANT) or from above (SYN). The standard phototropic setup (STA) was done on vertically positioned culture flasks with a laterally positioned BL source. Lateral displacement studies (LAT) were a variant of the STA setup, with flasks placed on a simplified turntable enabling displacement of culture flasks in front of a fixed BL source. The bending process was documented by Nikon Coolpix P510 and P520 cameras under a 5 min time-lapse regime. ANT, SYN and STA setups were imaged from the side and LAT from a vertically positioned camera through a transparent foil replacing the polypropylene closure.

Two culture flasks each with six shoots in a hexagonal arrangement and a separate BL LED lamp could be simultaneously mounted as a tandem for PT stimulation. For LAT setup studies, up to five plantlets per flask were arranged in a line. Tandem treatments were replicated 3–4 times, providing no fewer than 30 plantlets per treatment. Explants that failed to elongate and those that manifested no bending response were excluded from measurements. Curvatures were measured from stored digital images using Gimp 2.8 (www.gimp.org). Graphic presentations of PT and GT bending comprising mean values and their standard errors were drawn with OriginPro 8 (www.originlab.com).

## Results

### Standard PT and GT bending setups

In the STA setup, plantlets in their culture vessels maintain the same vertical position they had through the subculture. Horizontally positioned light from a BL source mimics early morning light in nature, and GT stimulation is absent until shoot tips start to bend toward the BL source. In the STA setup, PT bending exhibits a sigmoidal graphical presentation, with a point of inflection roughly at the midpoint between the starting and ending shoot positions. The starting position of vertically aligned shoots is considered zero, and bending stops when shoots reach ~90° deflection and align with the light source. However, the final 90° bending angle is reached only in morning PT treatments. In those starting later in the afternoon and at night, shoots are unable to reach the full bending, regardless of the time available for bending (Vinterhalter et al. 2015). GT stimulation in the STA setup cannot be prevented.

Standard GT bending was obtained by simple overturning of culture vessels with plantlets from the vertical to horizontal position. GT treatments were done in darkness, excluding the possible influence of PT bending. Therefore, standard GT bending provides clean results, unaffected by PT bending. In the standard setup, GT bending has a somewhat slower start, with a 15–20 min longer lag phase in comparison to the standard PT bending (Vinterhalter et al. 2015).

### Synergistic setup

**Fig. 2:**
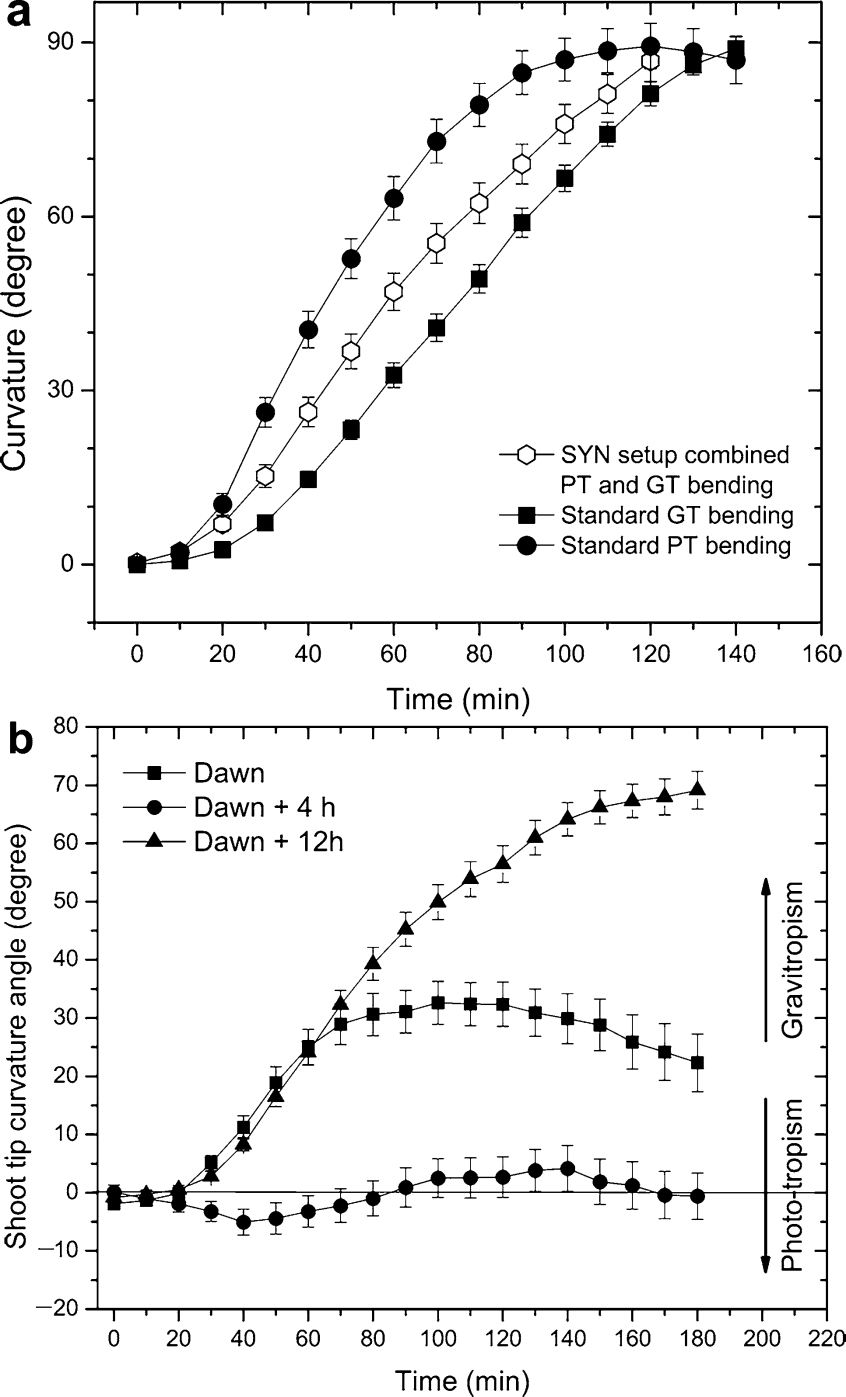
**a** In the synergistic setup position (SYN), phototropism and gravitropism join forces to bend the shoot upward. **b** In the antagonist setup position (ANT), the two tropic forces work against each other, and the outcome is variable depending mostly on the time of day. SYN and ANT graphs present mean values for shoot tip curvatures ± standard error,* n* ≥ 30 shoots per treatment

The SYN setup is a situation frequently encountered in nature as it occurs when plant shoots get in the horizontal position. In this case the PT and GT bending forces join their efforts to bring shoots back to their normal, vertical position. Thus for the potato shoots mounted in SYN setup there was a single and unambiguous bending outcome with all shoots quickly bending upward (Fig. 2a). However, although working synergistically PT bending in this setup was not just a mere addition of individual PT and GT contributions. On the contrary it resembled more the average of the two bending responses.

### Antagonistic setup

In the ANT setup, tropic forces are arranged to confront each other. PT bending is expected to bend shoots downward, while the GT bending works to restore the normal, vertical shoot orientation. However, the outcome of ANT setup treatments was highly variable, depending mostly on the time of the day when the experiments were started. Only during the period of the morning PT maximum were PT and GT bending balanced, with phototropism only slightly dominant at the employed BL irradiance. At all other times of the day (during the night, at dawn and late afternoon), gravitropism was the dominant tropic force, with plants producing a clear upward bending GT movement.

At dawn all shoots demonstrated a strict initial GT bending. After 50 min GT bending started to decrease turning after 100 min into a phototropic bending response (Fig. 2b). Phototropic bending developed only in the apical shoot portion providing a charcateristic S-shape to affected shoots.

**Fig. 3:**
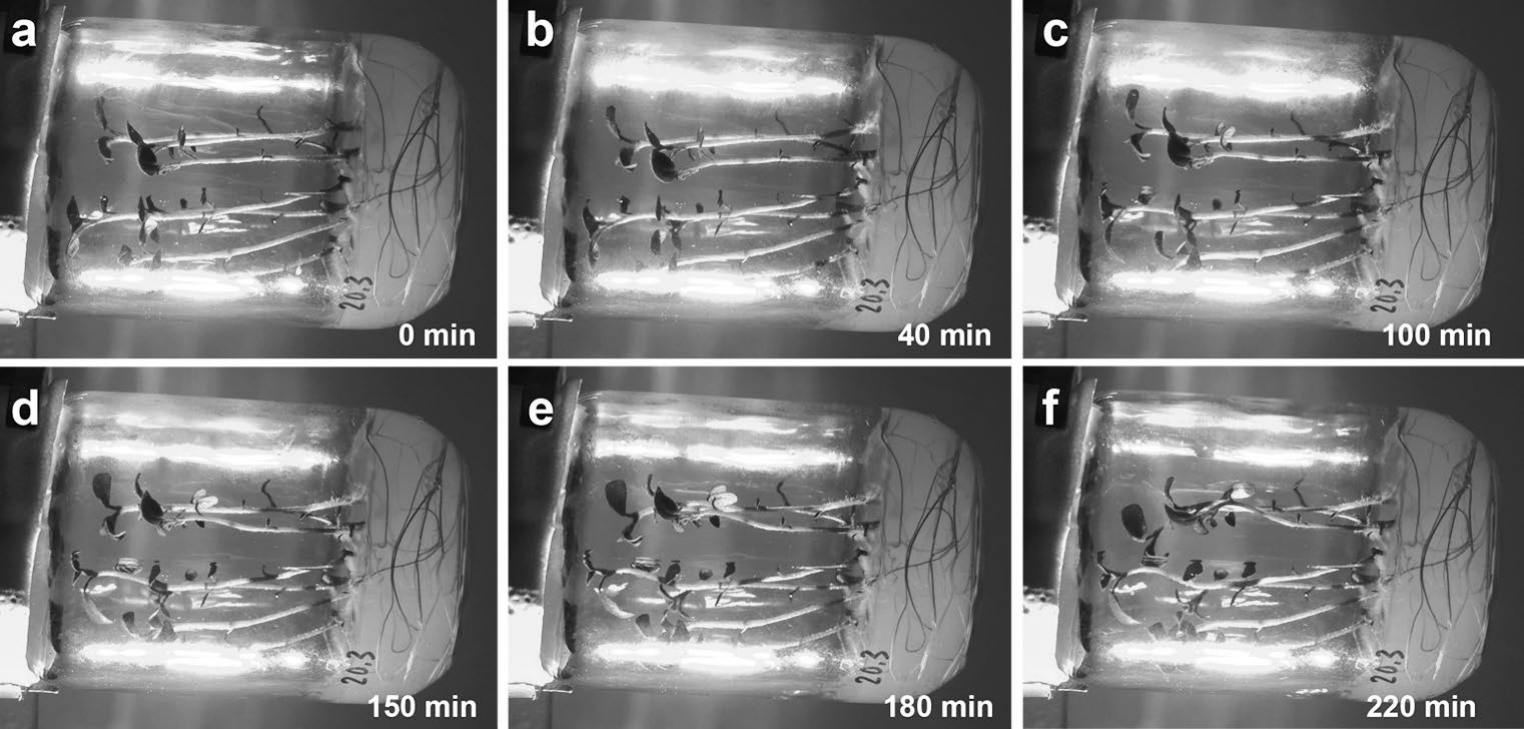
Formation of S-shaped shoots in a culture vessel with plantlets mounted in ANT setup position started 4 h after the dawn. Culture vessel diameter 62 mm

In treatments started 1–4 h after the dawn (08:00–10:00 a.m.), PT and GT bending responses were clearly antagonistic. Most plantlets initially had a small PT response visible after the first 30**–**40 min of the ANT treatment. The response consisted of downward bending of the shoot tips toward the BL source. Some 45 min from the beginning of treatment, bending changed direction, becoming somewhat GT until a small maximum at 140 min was reached, at which point the response then again became PT. Bending magnitudes on average were small, barely visible on the graph–first some 3.5° downward from the horizontal as the PT response and then some 4° upward in the GT direction (Fig. 2b). At the end of 3-h-long ANT treatments, the average shoot response was PT, with the average bending angle of just 1.3°. One-third of the plantlets manifested a gross GT bending response. Among deviations were plantlets with S-shaped shoots. Their apical shoot portion was PT, but a second bending position some 20 mm from the shoot tip created a middle shoot portion, which was gravitropic (Fig. 3). S-shaped shoots are an important observation, as they indicate that even in the same shoot, adjacent shoot portions can exhibit different tropic responses.

The slow initial establishment of the tropic response and differences in the zones of the highest tropic responses along the shoot clearly indicate that PT and GT bending in the ANT position are independent and concomitant processes. Working constantly as antagonists, the processes prevent each other from establishing proper, sigmoidal tropic curvatures characteristic of standard PT and GT bending conditions.

**Fig. 4:**
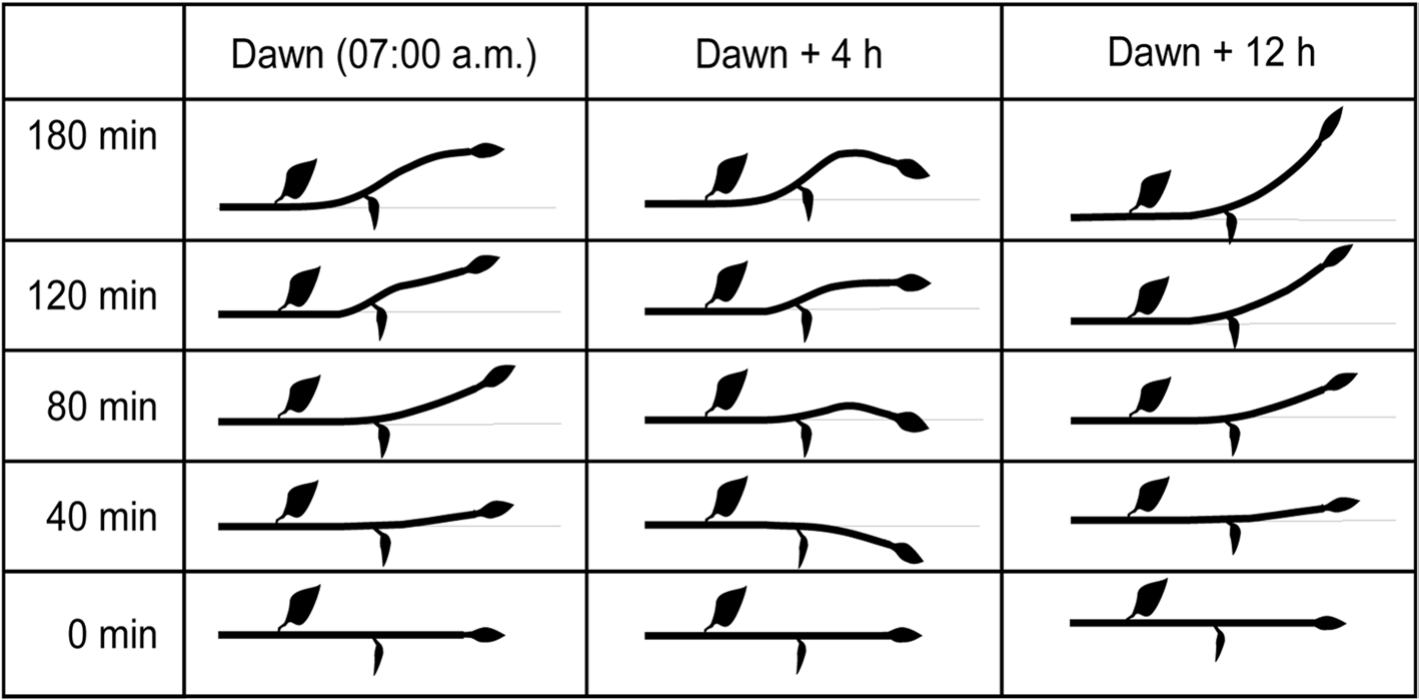
Shematic presentation of shoot tropic bending in the ANT position mounted at three different times of day (dawn, dawn +4 h and dawn +12 h). Shoots are initially in* horizontal position*. Downward bending denotes phototropic (PT) and upward bending gravitropic (GT) response of shoot tips and middle segments

In treatments started 6–8 h after dawn (13.00–15.00 h), corresponding to the end of the morning PT maximum, the initial gross PT response gradually changed during the treatment into a GT response. In the experiments started 12 h after the dawn (Fig. 4) same as in those started during the night period, the GT bending of cultures was dominant from the beginning until the end of treatments.

ANT setup experiments indicate diurnal changes of tropic competence (Vinterhalter et al. 2015) as the major factor controlling the gross tropic response of cultures. PT and GT bending forces exert their effects simultaneously from the beginning of treatments, but their overall (gross) tropic shoot response is apparently a result of interaction of these forces and can significantly change throughout the day (Fig. 4). However, the ANT setup is a situation not likely to be encountered by plants in nature, and, therefore, its findings are of limited concern.

### Subdued GT activity

The STA setup still holds the question of subdued GT activity. A problem is that with the progress of vigorous PT bending, the expected GT bending of this setup appears to be subdued, cancelled or even totally absent. Indeed, at the beginning of PT stimulation, the GT bending of the vertically positioned shoots is not yet induced (triggered). With the progress of PT bending, GT stimulation increases, but there is no visible GT bending response. Our previously presented ANT studies indicate that the GT bending force is always present, even if its manifestation is not always visible (expressed). Thus, in the STA setup, PT seems to balance and counteract GT bending all the time. If this is a correct assumption, then turning the BL off (transient darkness) would break this balance and trigger GT-assisted straightening.

**Fig. 5:**
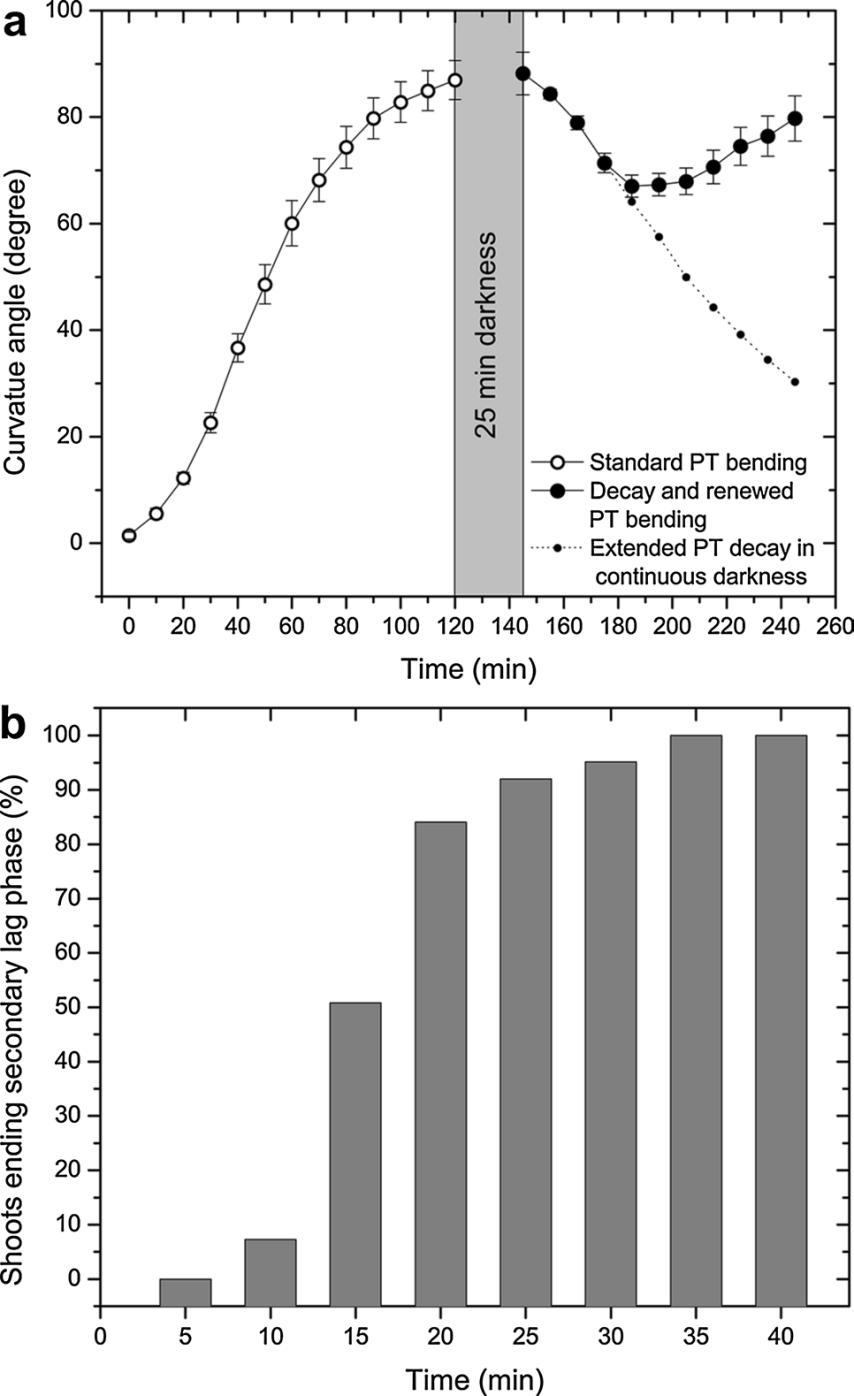
**a** Phototropic (PT) bending in the standard setup position subdues and prevents manifestation of the gravitropic bending. At the end of 120 min PT bending, plantlets can maintain the obtained* horizontal shoot position* for hours. However, if the blue light (BL) is turned off, then PT bending decays as shoots enter straightening. Turning the BL on after 25 min of darkness restarts PT bending but only after a new lag phase.* Graphs* present mean values ± stardard errors,* n* ≥ 30 shoots per treatment, **b** Phototropic (PT) bending in the lateral displacement setup induces a secondary lag phase after lateral displacement of the light source. The *figure* presents percentages of shoots ending secondary lag phase and entering lateral PT bending after 90° light displacement at 5 min intervals,* n* = 63 plantlets

Straightening does occur when the BL is turned off at the end of STA treatments. After 20 min of darkness, the first signs of straightening are observed, and straightening progresses with increased duration of darkness. However, turning the BL on again induces a new round of PT bending which recovers its dominant role, overriding the GT bending response that started in the darkness (Fig. 5a).

Data presented in Fig. 5a show that after a 25-min-long period of darkness, the PT bending force decays and shoots require a new lag phase before they can commence and complete PT bending. Tropic bending effectors are far from being exhausted by a single tropic bending event, and shoots during the morning PT maximum are ready to perform multiple PT bending events. However, every new PT bending event requires a new lag phase, regardless of whether it followed a change in light direction or a mere renewal of PT stimulation after the previous round of PT bending expired.

We have seen that in the STA position the early start of PT stimulation prevents the expression of later-started GT stimulation. The reverse type of this experiment, with an early start of GT stimulation followed by later PT stimulation, can be arranged in the ANT setup. In this case, BL stimulation applied 30–60 min after the beginning of GT stimulation appears to suprres PT bending. Thus it seems that the first established type of tropic response bending subdues and prevents later expression of the other response. But in case of later started PT stimulation suppression results not from the activity of GT bending but simply from the prolonged lag phase duration of PT bending induced by darkness (Fig. S4).

### Horizontal adjustment of shoot position

The existence of a second lag phase prior to the renewed PT bending in Fig. 5a is difficult to notice and prove, as it appears just as a delay before the new round of PT bending. However, if the light source in the STA-setup PT bending is laterally moved to another position (LAT setup), then the second lag phase will become visible as the shoot abandons the original vertical plane of bending, making new spatial adjustments in the horizontal plane. A vertically positioned camera can follow this horizontal shoot movement, and time-lapse photographs can be used to measure the rate of lateral shoot adjustment. The change of light source position occurring in the LAT setup does not affect the GT stimulation of plantlets, and, therefore, all subsequent responses appearing in this setup can be attributed only to the PT bending (clean response). Hence, changes in the bending direction can be explained only as the redistribution of the PT bending force.

**Fig. 6:**
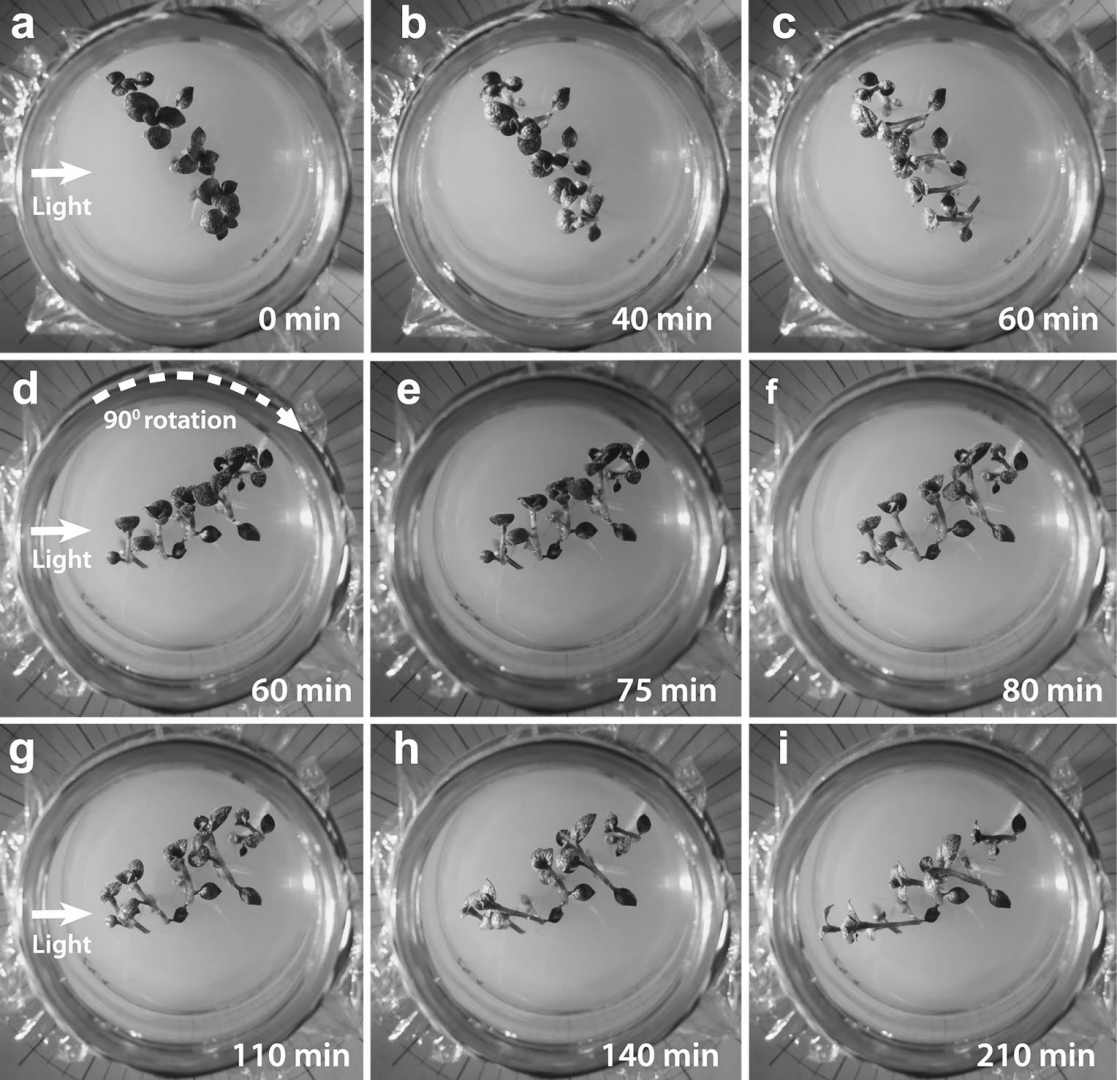
Lateral displacement setup position bending of a group of plantlets in their culture vessel toward blue light (BL) coming from the* left side* (*arrows* in **a**, **d** and **g**). Following the initial lag phase, shoots bend to the* left* (**b**, **c**), but after 60 min, the turntable with the culture flasks is turned 90° clockwise (**d**), starting the secondary lag phase. For the first 15 min after lateral light reallocation, (**e**) shoots bend by inertia in the wrong direction, but 5 min later (**f**) they start to correct their position until they again get aligned with the new BL source position (**g**, **h**). Culture vessel diameter 62 mm

In the LAT setup position, treatment shoots were first allowed to bend in a vertical plane 55–60 min toward a light source, which was then laterally shifted (displaced) in the horizontal plane to some other position, keeping the same distance from plantlets. Although the LAT setup is just an STA position variant, there are some important differences. PT bending in the LAT setup is composed of two stages separated by an additional (secondary) lag phase (Fig. 5b). After the initial lag phase, there is first a vertical PT bending stage (component) equal to STA-position PT bending, which brings shoots from a vertical to a more horizontal position, facilitating determination of the bending direction. Horizontal change of light source position instantly triggers a new, second lag phase, during which the vertical PT bending continues by inertia, slowing down until the end of this lag phase. The second stage of LAT-setup PT bending is composed of horizontal (lateral) shoot bending, adjusting shoots to the new position of the light source (Fig. 6).

**Fig. 7:**
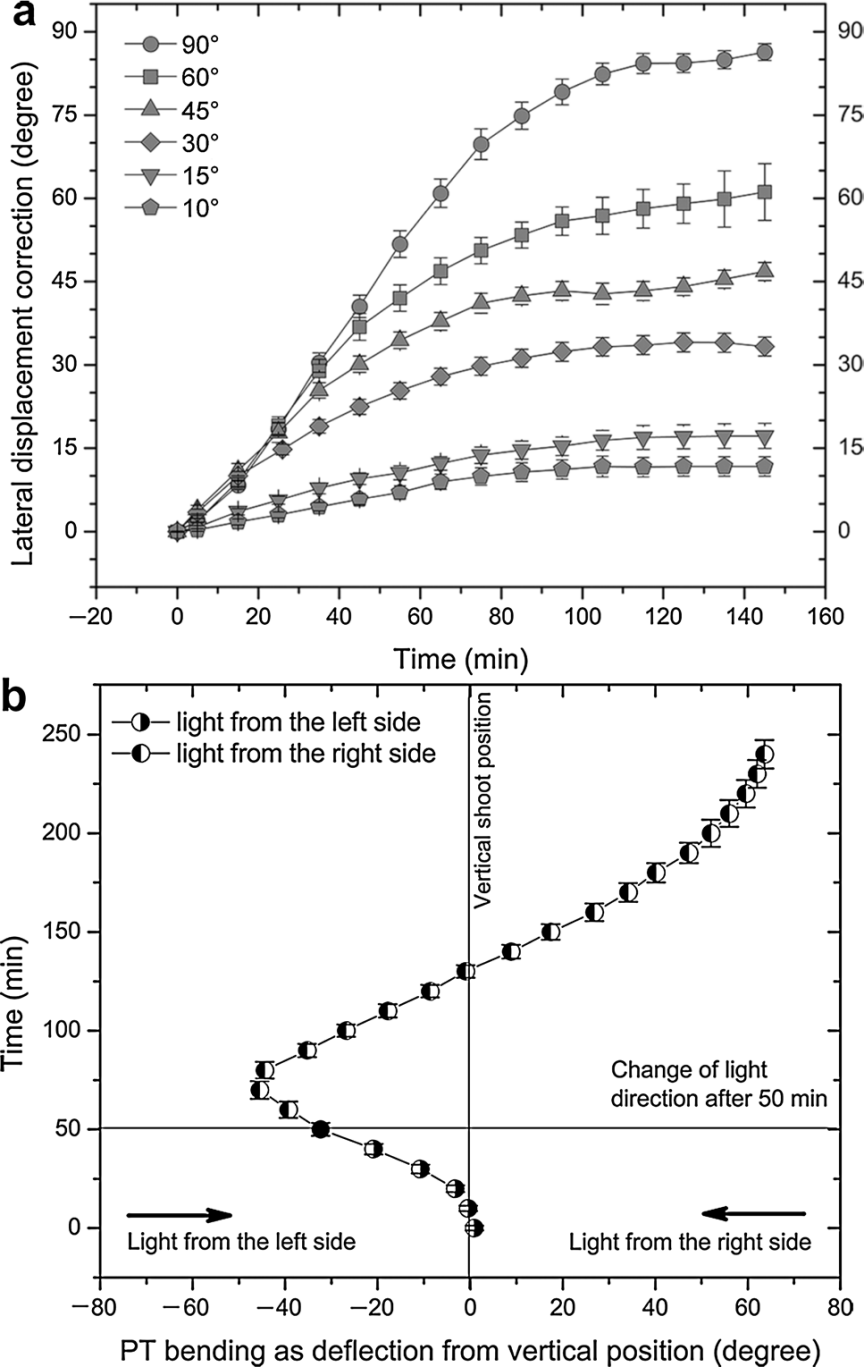
**a** Following lateral displacement of the light source, plantlets adjust the spatial position of their shoots by phototropic (PT) bending, both in the *vertical* and *horizontal planes*. The graph presents rates of horizontal position corrections after 10°, 15°, 30°, 45°, 60° and 90° angle displacements of the BL source following a 60-min long standard position setup PT bending pre-treatment. Zero at the X axis is time of light source lateral displacement. Points in the graphs are mean values of shoot tip lateral angle correction ± stadard error,* n* ≥ 30 shoots per treatment, **b** In the reversion setup position, phototropic (PT) bending was first done for 50 min toward the blue light (BL) source on the left, which was then displaced horizontally to the opposite right side (180°). A secondary lag phase appeared lasting until the shoots started to revert their bending direction. Bending to the second light source continues in the same vertical plane but in the opposite direction. To differentiate the two PT bending stages presented in the graph, REV bending was shown with *negative* (–) values for bending to the first (*left*-positioned) light source and with *positive* (+) values for bending to the second (*right*-positioned) BL source. Graph points are mean values for shoot tip curvatures ± standard error,* n* ≥ 30 shoots per treatment

The LAT setup position studies showed that the PT bending force can be partly or completely redistributed from a vertical into a horizontal component. The rate of lateral adjustment and redistribution ratios both depend on the light source displacement angle. The rate of horizontal bending increased with the displacement angle (Fig. 7a). The rate of horizontal bending although variable was sufficient to compensate 10–90° lateral displacement of the light source in some 120–150 min. At the highest 90° angle, the horizontal bending component had a rate closely resembling the vertical PT bending rate of shoots in the STA position.

Redistribution of PT bending appears to be more complex, as it depends both on the displacement angle and time of displacement. At lower displacement angles (up to 30°), part of the PT bending force is detached for lateral bending, while the vertical bending component still goes on without delays; there is no lag phase for the vertical bending component. At higher displacement angles, a faster lateral bending rate indicates that a larger ratio of PT force is detached for lateral adjustment and that vertical bending is usually arrested. At still higher displacement angles (greater than 60°), the remaining vertical component following redistribution was not sufficient to balance the GT bending, and these shoots entered transient straightening, as shown in Fig. 5a. Once the horizontal shoot position was adjusted, the vertical bending reappeared, finishing the vertical PT bending component. If the light source displacement is done in early stages of PT bending before the shoot has reached the point of inflection, then the lateral correction is performed in less time, enabling shoots to quickly commence and finish the vertical bending component.

**Fig. 8:**
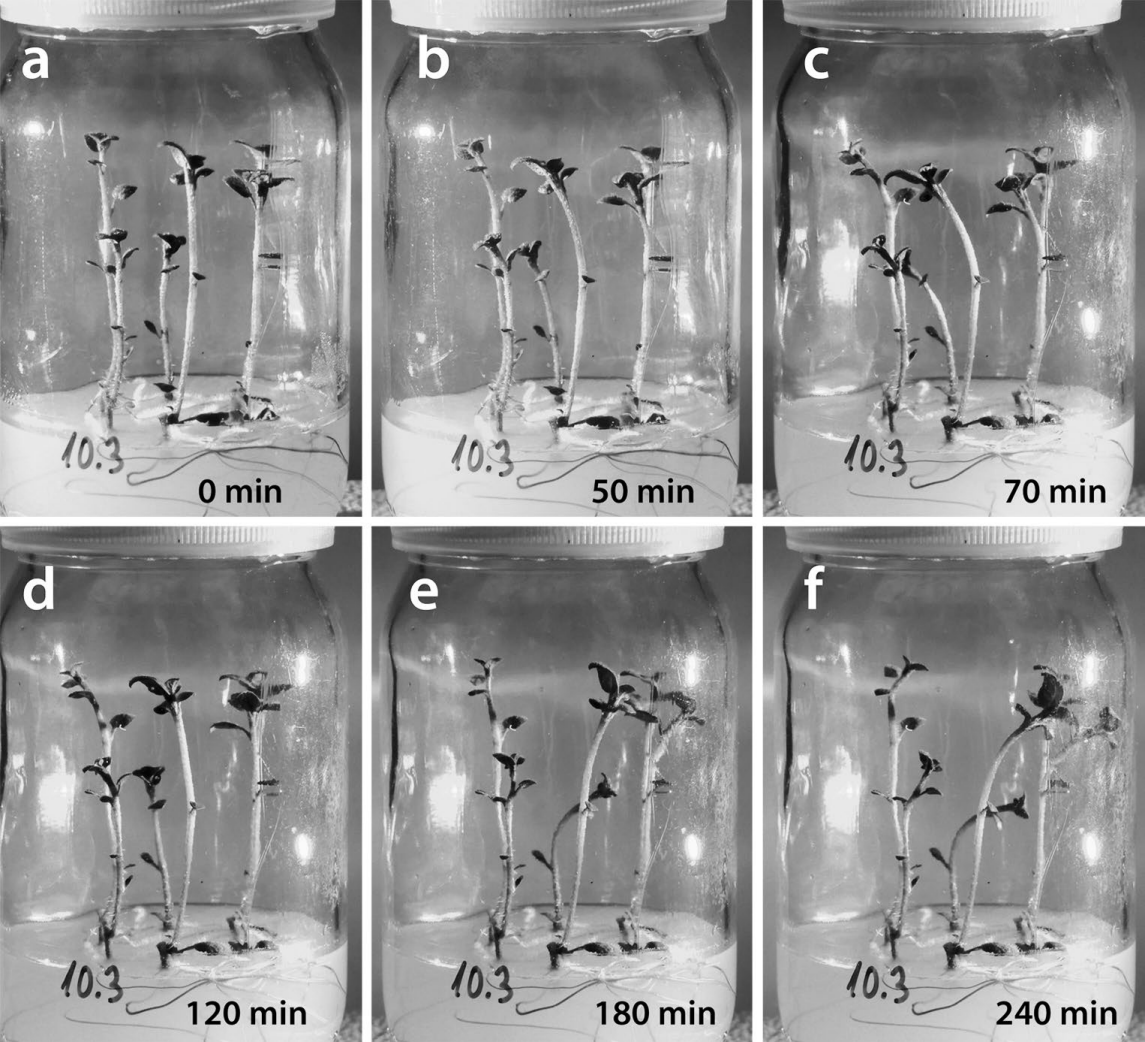
Phototropic bending (PT) in a culture vessel with plantlets mounted in the reversion setup position. Plantlets were bending first to the left side (**a**, **b**), and then 50 min later the blue light source was moved to the opposite (*right*) side. For the next 20 min, corresponding to the secondary lag phase, **c** shoots continue to bend to the left by inertia, and then they revert their bending direction (**d**–**f**), entering a long stretch of linear bending. Culture vessel diameter 62 mm

The REV setup is a special case of the LAT setup that occurs when the BL light source is displaced exactly 180°, and it results in complete reversion of the shoot bending direction (Fig. 7b). Bending before and after light source displacement occurs in the same vertical plane, and, therefore, there is no horizontal component of PT bending. A second lag phase is interpolated, and its presence is visible as PT bending slows down until it stops and then restarts in the new (opposite) direction. Thus, potato shoots in full swing of PT bending can rapidly revert shoot bending into the opposite direction (Fig. 8).

In contrast to the STA setup, which has a very short linear phase of PT bending around the point of inflection, the REV setup has a more than 80-min-long stage of steady, linear PT bending. Thus, although the shoot tip follows an arc in its bending, the bending rate itself is linear. Total angle that shoot tips covered during the PT bending in the REV setup was ~108° and that was significantly higher compared to the ~90° bending of the STA setup (Fig. 2a). Therefore, it seems that classic STA setup treatments are just too short to present the full scope of PT-bending ability of potato shoots. However, at the end of the REV setup bending shoots are not aligned with the BL source as they reach average bending angle of just ~64°.

Studies with a camera mounted above the culture flasks during the LAT position studies revealed an anomaly at the beginning of PT bending. Initially, shoots were not accurate in position determination, and after 60 min they only roughly bent in the direction of the light source. Error seems to arise from internal constraints of potato shoots. However, fine shoot positioning completely correcting this error occurred in later stages of PT bending. It is noteworthy that shoots performing lateral shoot position corrections were prone to overshoot the new light position at all except the highest 90° displacement angle.

## Discussion

PT and GT interactions have been studied in a number of light-grown or de-etiolated plant species, including sunflower (Franssen 1980), cress (Hart and Macdonald 1981) and *Arabidopsis* (Fukaki et al. 1996a, b). In these species, gravitropism was the dominant tropic response, overpowering the PT response. Complex tropic response interactions together with multiple PT responses were shown to exist both in *Arabidopsis* (Sato et al. 2014) and potato (Vinterhalter and Vinterhalter 2015).

In potato shoot cultures grown in conditions of a long-day photoperiod (16/8 h), gravitropism was dominant only in certain periods of day, including dawn, late afternoon and night hours. Morning and midday hours (08:00–15:00 a.m.) comprised the period of strong PT competence in which BL, if provided at sufficiently high irradiance, counteracted GT bending.

According to our ANT setup studies, PT and GT bending are concomitant but independent events. If PT and GT stimulation start at the same time, then GT bending is not subdued and PT versus GT bending ends in a rough balance, indicating that they compete for the same effector mechanism. Higher BL irradiance can tip this balance in favour of PT bending, while lower irradiation favours GT bending. However, suppression of the GT bending response occurs only if PT bending starts first, as in case of the STA setup. Results presented in Fig. 5a show that once PT subdues GT bending in the STA setup, then PT bending can regain its dominant role in shoot bending even after a transient cessation of PT bending.

In a previous study (Vinterhalter et al. 2015, Fig. 3) we showed that a period of darkness interpolated before the beginning of PT stimulation significantly affected PT bending prolonging the lag phase duration. Short up to 20 min long periods of darkness passed unnoticed, but a 60 min long period doubled the lag phase duration. Darkness on the other side did not affect the lag phase duration of GT bending and it even somewhat improved its magnitude. Thus in the ANT setup treatments with delayed beginning of PT stimulation darkness apparently prolonged the lag phase duration of PT bending. Hence the observed suppression of PT response here seems to be just a delay in the PT bending response supporting the independant nature of tropic bending responses.

Formation of S-shaped shoots has deep implications, as it shows that the locations of highest PT and GT bending along the shoot do not overlap. PT bending is located close to the shoot tip (10 mm), while GT bending is more widely dispersed along the shoot. Decreases in the BL intensity of the light source, shade from a large leaf thrown on the shoot or even liquid layers accumulating in the lower part of culture vessel dispersing the incoming light can all locally tip the PT versus GT balance and promote the appearance of S-shaped shoots. However, the important point here is that neighbouring segments of the same shoot can manifest radically different tropic responses.

Findings from SYN setup treatments indicating that contributions of PT and GT bending are not additive complete the picture of potato tropic interactions, in which PT and GT forces have different inputs but a common output, as they probably address the same effector. Apparently, a tropic response effector has a limited capacity that quickly gets saturated.

LAT position studies clearly showed existence of secondary PT bending lag phases, in which bending shortly continues by inertia toward the original position of the light source. In the primary lag phase of all setups, PT bending always started abruptly from a zero point. However, there are some exceptions, as in case of plants grown in continuous light (Vinterhalter and Vinterhalter 2015), where a short 45–60 min long period of darkness triggered circumnutation-like movements. Since plants at the beginning of PT stimulation following this treatment were already bending in some direction, we had to change the methodology of lag phase determination. Instead of extrapolating the average process time to reach the 10° bending angle (Vinterhalter et al. 2015), we now determine the time of the first observed tropic movement for each plantlet in 5-min interval increments (Vinterhalter and Vinterhalter 2015).

The ability of shoots to equally well adjust their position in both the vertical and horizontal direction (Fig. 8a) was somewhat surprising. However, the key conclusion from LAT position studies was that PT bending can get partly or completely redistributed to other directions. The camera positioned above the culture vessel registered not only direction in which the apical shoot portions were bending but also the length of that segment. From these data, it was possible to measure the rate of horizontal shoot position adjustments and to estimate the shoot bending rates before and after the lateral displacement of the light source.

As there is no change of direction or intensity of GT stimulation in the second stage of PT bending, there is no new force to confront and stop the horizontal component of PT bending apart from the general slowdown of PT bending resulting in overshooting. For this same reason, solar tracking with constant maintenance of a steady horizontal bending rate (Vinterhalter and Vinterhalter 2015) has a pronounced inertial bending at the end of PT stimulation.

Attempts to determine the threshold angle for the horizontal light source displacement that induced a secondary lag phase were not successful. This was probably caused by the initial inaccuracy of shoots in the determination of incoming light direction combined with overshooting. If lateral displacement angles are sufficiently small (as in case of sun-tracking studies (Vinterhalter and Vinterhalter 2015), then these small lateral light source shifts get ignored, and, as there are no secondary lag phases, PT bending proceeds smoothly.

The concept of virtual tropic bending forces can be useful if we try to imagine events that are going on in real time during the bending process. Tropic movements have their preferential shoot halves, in which cell elongation is stimulated, leading to shoot bending; for phototropism, it is the shaded half of the shoot (Orbović and Poff 1993), and, for gravitropism, it is the lower shoot half (Masson et al. 2002). In the ANT position, each force develops in its own preferential shoot half counteracting the effect of the other one. In the SYN position, both forces are in the same preferential side. However, the number of effectors is limited either way and, therefore, their combined effect cannot be additive. In the STA position at the beginning of PT bending, there is no GT stimulation, and by the time it should start to develop, the effector system is already occupied (recruited), providing PT directional bending that can be changed only if the BL is turned off for some time or if the PT bending competence expires following the changes in diurnal rhythms. In the LAT position, the change of illumination to the lateral side induces redistribution of the PT bending force into the lateral direction, creating an asymmetry in the distribution of tropic forces within the shoot. This asymmetry creates a torque momentum force responsible for the previously observed shoot torsion twisting (Vinterhalter and Vinterhalter 2015).

We can imagine virtual tropic forces as a transient asymmetry in the radial flow of water within the shoot induced by PT and/or GT stimulation. To fit the expected response, water flow needs to be inhibited on the BL-irradiated side during PT bending and on the upper shoot side during the GT response. Asymmetric changes of the radial water flow are sufficient to explain all observed PT versus GT interactions, lag phase characteristics and rapid changes in bending directions of potato shoots. These responses indicate also how PT and GT shoot bending compete for the water translocation resources. Finally, the strong suppression of the GT response by PT bending in the STA setup and similar delayed PT bending in darkness of the ANT setup can be explained with the well-known ‘first come–first served’ principle.

In spite of the findings presented here, we recommend that studies of plant phototropism should be extended to cover other setups and interactions in addition to the standard PT and GT bending studies. The STA position setup follows only the vertical component of PT bending, and it does not accurately present daily events that plants encounter in their natural surroundings, neglecting the horizontal bending component and, therefore, the true bending potential of the investigated species.

## Electronic supplementary material

Below is the link to the electronic supplementary material.

Supplementary material 1 (PDF 749 kb)
