# Supplementary material for: Interaction with gravitropism, reversibility and lateral movements of phototropically stimulated potato shoots
Source: J Plant Res. 2016 Mar 31;129(4):759–70. doi: 10.1007/s10265-016-0821-4 (PMC4909813; doi:10.1007/s10265-016-0821-4)
Supplement: Supplementary file 1 — Supplementary material 1 (PDF 749 kb) [file 10265_2016_821_MOESM1_ESM.pdf]

Electric Supplementary materials

Title:

Interaction with gravitropism, reversibility and lateral movements of phototropically stimulated potato shoots

Authors:

Vinterhalter D, Savić J, Stanišić M, Jovanović Ž, Vinterhalter B

Journal:

Journal of Plant Research

Corresponding author

Vinterhalter Dragan

Institute for Biological Research, Plant Physiology Department, University of Belgrade,

Bulevad despota Stefana 142, 11060 Belgrade, Serbia

phone 381 1 2078428, fax 381 1 761433

dvinterhalter@yahoo.com vinter@ibiss.bg.ac.rs

Content:

Fig. S1

Fig. S2

Fig. S3

Fig. S4

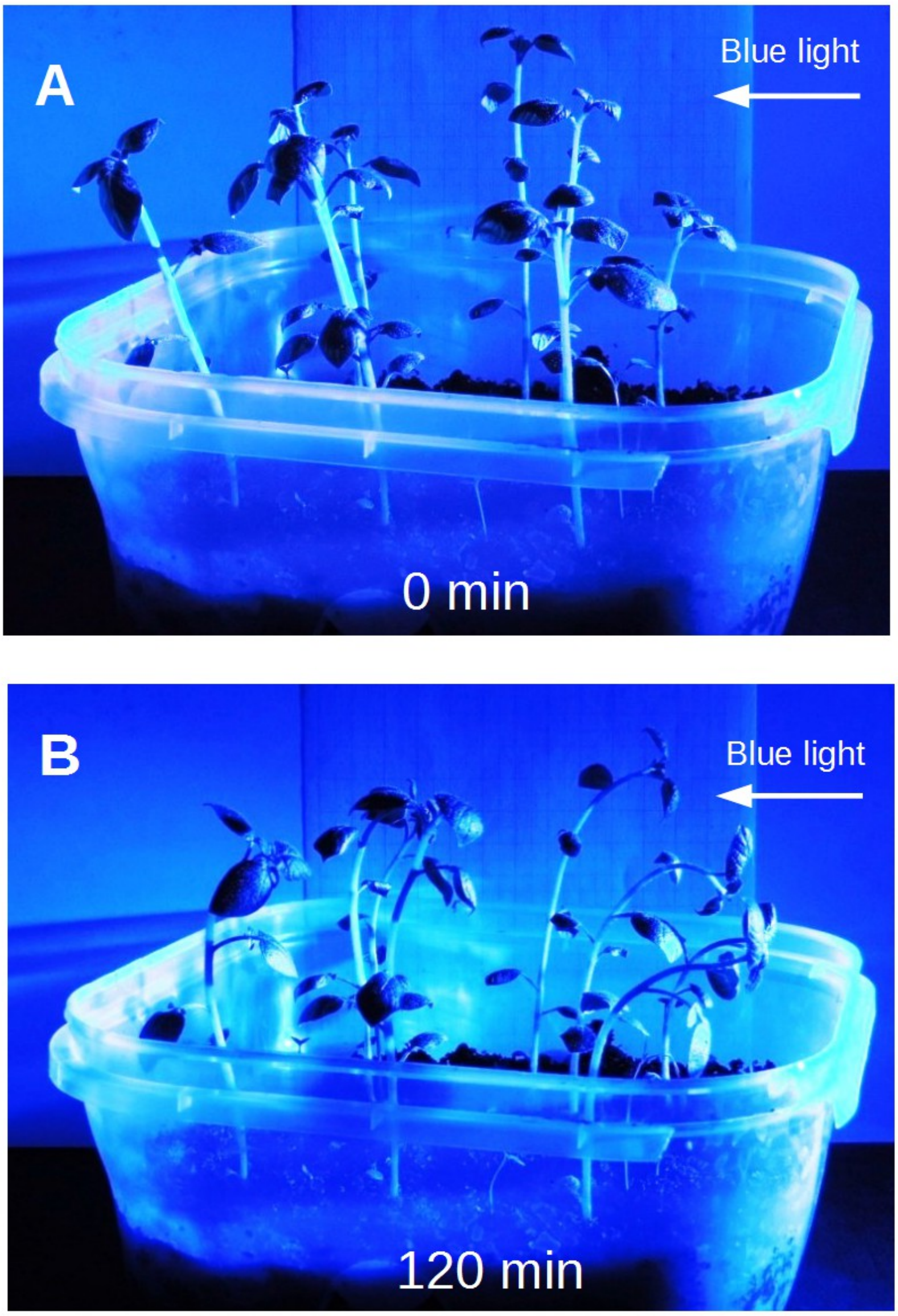

Fig. S1
Phototropic bending (PT) of *in vitro* cultured potato plantlets acclimated for 30 d in a shaded glasshouse, (A) at the beginning of phototropic (PT) stimulation, (B) after 120 min of phototropic (PT) stimulation in the standard (STA) position setup

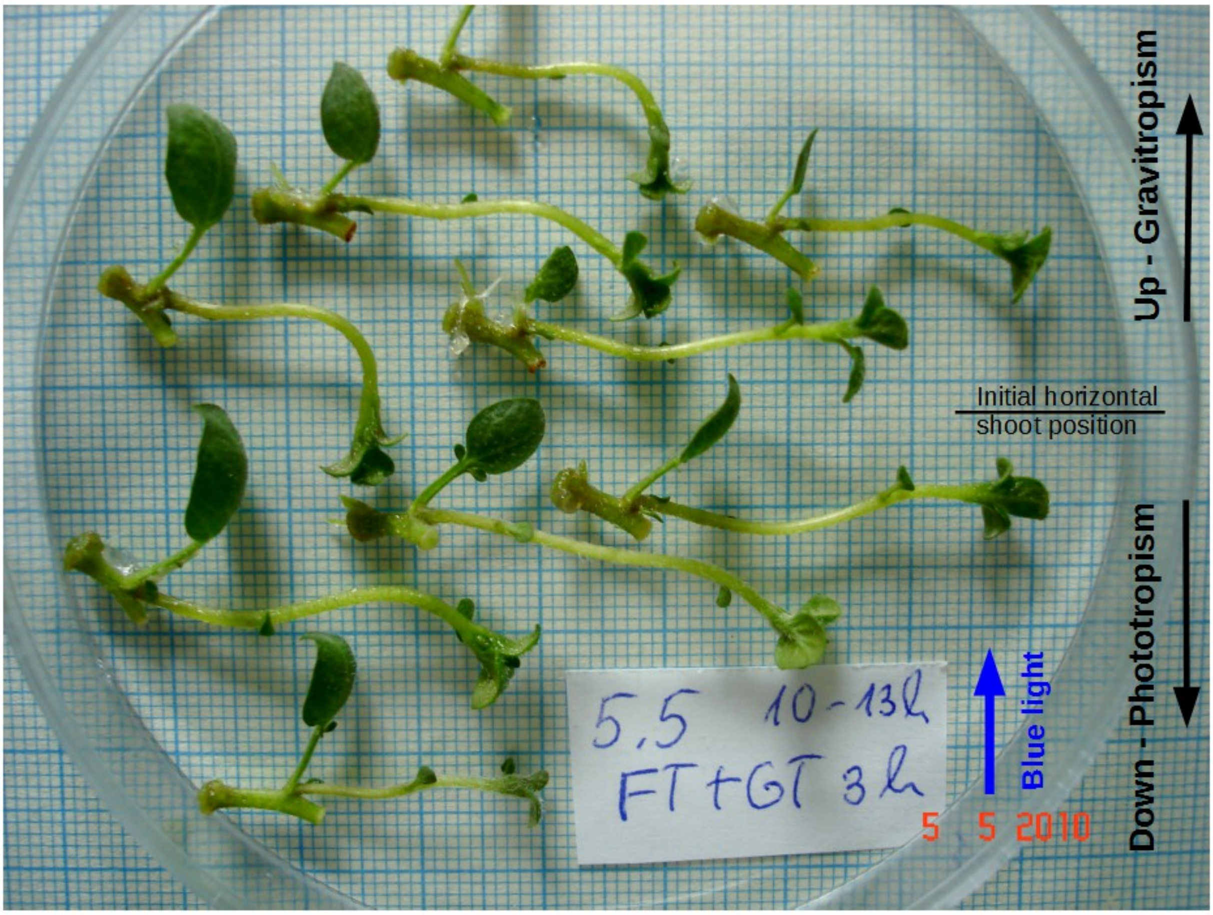

Fig. S2
Plantlets from ANT setup treatment with S-shaped shoots. Shoot tips are preferentially phototropic (PT) bending downwards towards the inc oming blue light but the middle shoot portions bending upward are gravitropic (GT)

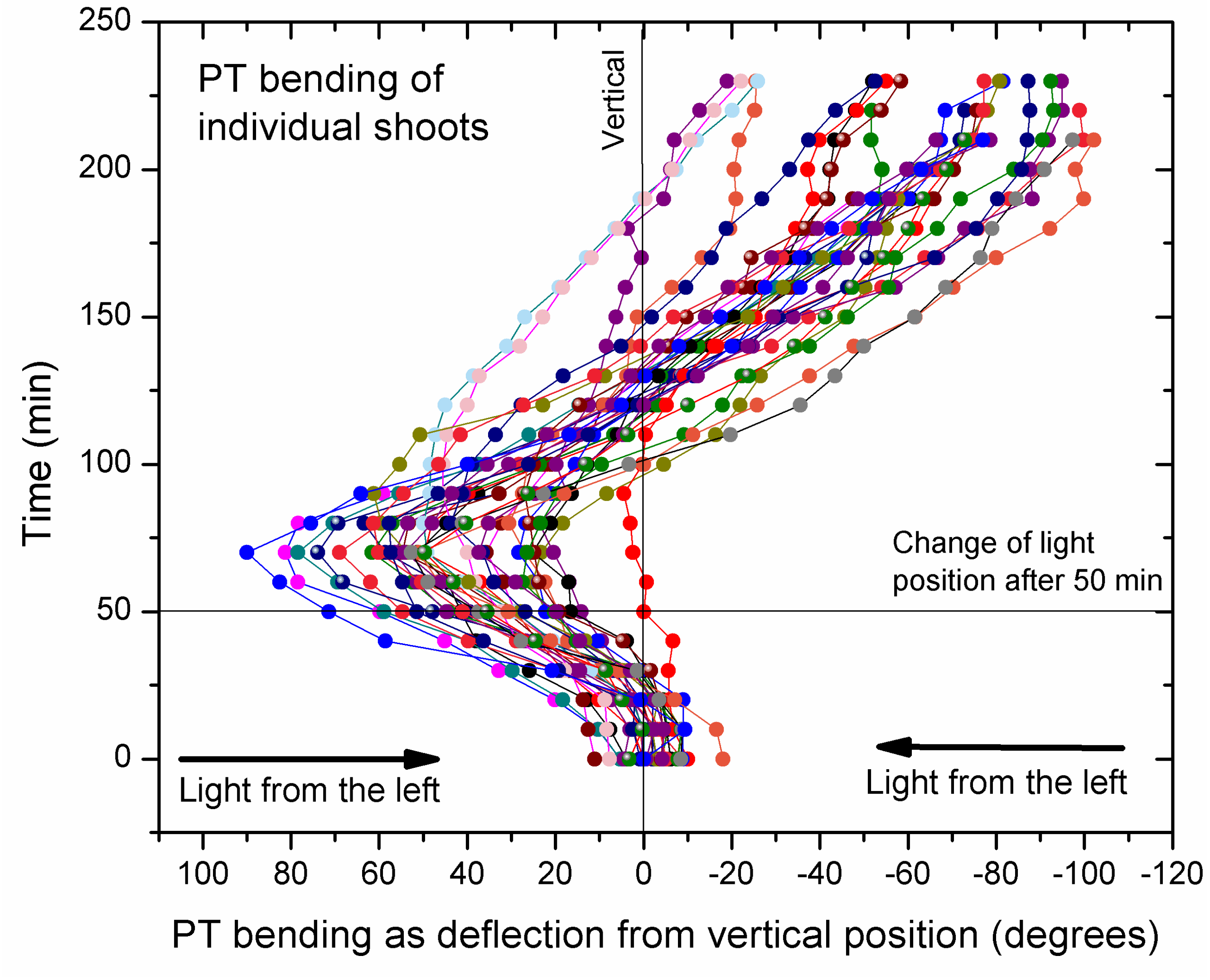

Fig. S3
Reversibility of the phototropic (PT) bending direction is a prominent feature of potato shoots. Change of the light position in REV setup provides good response synchronization with little variability among individual plantlets presented here. Their average PT response is presented in Fig. 7b

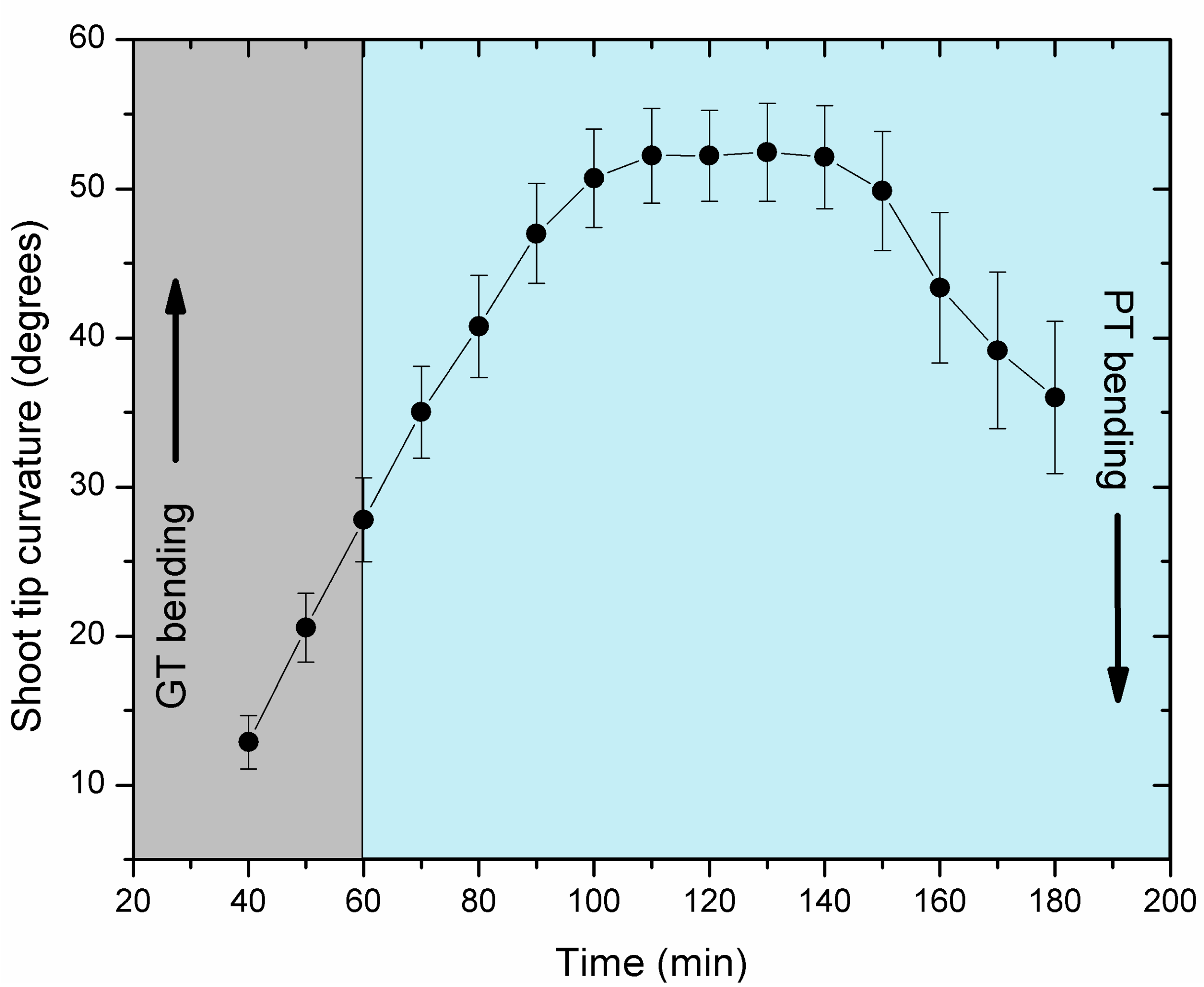

Fig. S4
ANT setup position in which blue light is turned on after 60 min providing GT bending with a 60 min lead, suppressing the PT bending response. Duration of the PT bending lag phase is prolonged to more than 30 min instead of 15 min characteristic for periods of morning PT bending maximum. Delayed PT bending seems not to be a result of direct GT action but rather of prolonged lag phase duration induced by a darkness pretreatment as previously shown (Vinterhalter et al. 2015, Fig. 3a)
